# Electrospinning of *Miscanthus x giganteus* Organosolv Lignin in Dimethyl Sulfoxide (DMSO)

**DOI:** 10.3390/polym17121695

**Published:** 2025-06-18

**Authors:** Roland Jacks Ekila, Tatjana Stevanovic, Denis Rodrigue

**Affiliations:** 1 Department of Wood Sciences, Faculty of Forestry, Laval University, Quebec, QC G1V 0A6, Canada; roland-jacks.ekila.1@ulaval.ca; 2 Department of Chemical Engineering, Laval University, Quebec, QC G1V 0A6, Canada; denis.rodrigue@gch.ulaval.ca

**Keywords:** electrospinning, *Miscanthus x giganteus*, organosolv process, dimethyl sulfoxide, lignin, fibers

## Abstract

Electrospinning is a simple technique to produce fibers with small diameters. These fibers can be made from different polymers, but the focus is now on biobased materials. In this work, the lignin obtained from *Miscanthus x giganteus*, an herbaceous plant, was isolated by an Organosolv process leading to a high purity (90%), which is essential for its electrospinning. This lignin also had a carbon content of 72.2% with 24.8% oxygen and a low nitrogen content (1%). The isolated lignin was then solubilized in dimethyl sulfoxide (DMSO). Finally, an optimization step showed that a stable process was possible using a 62% lignin solution in DMSO with a needle-to-collector distance of 20 cm, a flow rate of 0.3 mL/h, a voltage of 25 kV, and a humidity of 35%. Nevertheless, lignin concentrations between 55 and 63% were studied to determine the effect of this parameter on the final fibers. A morphological analysis (SEM-EDX) enabled us to understand both the evolution of the diameter and the effect of dimethyl sulfoxide on the electrospun fibers. This study showed that electrospinning of the lignin obtained from *Miscanthus x giganteus* was possible, even without any additives.

## 1. Introduction

The use of fossil resources and their transformation have shown in recent decades a strong impact on the ecological state of our planet [1]. Due to their low weight, high resistance, and very small diameter, polymer fibers represent technological advances in many areas [2,3]. This innovation, made possible by the material’s spinning ability, is evolving rapidly thanks to the electrospinning technique [4,5]. Electrospinning can be used to produce thin and ultra-thin fibers which is essential for the development of finished products [6,7]. The process is based on electrical spinning using a high-voltage source to stretch a polymer solution into a thin fiber [8]. During the process, the polymer solution is injected through a needle to create a jet, which elongates thousands of times and consequently forms nano-diameter fibers with very high molecular alignment [3,9].

The electrospinning setup is composed of several equipment: a pump, a syringe, a collector, and a source of tension [7,10,11]. Synthetic polymers are often chosen as fiber-forming polymers. But natural polymers, such as cellulose and lignin, have recently attracted increasing interest [12,13,14]. While the formation of ultrafine fibers from cellulose was quickly tested, the discovery of lignin generated considerable interest, despite its branched and complex structure. It is very difficult to expect strong fibers from lignin, so this raises the question of what properties these fibers should have [3,10,15].

The electrospinning of lignin is favored by the presence of phenolic and hydroxyl groups, making lignin solutions more prone to electrolytic fields [16]. Lignin has other advantages including availability, low cost, biodegradability, and high carbon content [3]. But the complexity and fragility of lignin still pose challenges to developing 100% lignin-based fibers. Its heterogeneity and rigid structure also make this polymer difficult to extract with high purity [10,17].

Lignins differ not only by their origin, age, and location in the woody species, but also by their pre-treatment [18]. Lignins can be naturally found in three types: softwood lignins are mainly made of guaiacyl units, while hardwood lignin contains both guaiacyl and syringyl units, and herbaceous lignins are composed of guaiacyl, syringyl, and p-hydroxyphenyl units [3,18]. Wood, the most widely used source of lignin, has a content of over 20%, which can cause deforestation problems, hence the use of herbaceous plants, such as *Miscanthus x giganteus* [17,18]. *Miscanthus x giganteus* is a herbaceous plant composed of around 25% lignin made from 4% p-hydroxylphenol, 52% guaiacyl, and 44% syringyl units [19]. Lignins can be extracted through different pre-treatments, which can be divided into two groups: environmental (Organosolv, soda, and hydrolysis acid) and conventional (Kraft and sulfite) techniques [17,20,21].

The Organosolv process is based on organic solvents and is recognized as an eco-friendly, inexpensive, and non-polluting method [1,17,22]. Organosolv lignins are suitable for electrospinning because of their high purity, making them good candidates without modifications [23,24]. This work is based on an Organosolv lignin isolated by a process developed in-house. This process uses an ethanol/water mixture as the solvent and a Lewis acid-type catalyst (Fe_2_Cl_3)_. But the Organosolv lignin obtained by this process is slightly denatured compared to its native structure [18,24,25]. It is very difficult to electrospin pure lignin alone because the technique requires very good solubilization in an appropriate solvent [12,24]. The choice of a suitable solvent is a key factor influencing the properties of electrospun fibers [26]. Several solvents were proposed to electrospin lignins, most of them being used alone or as mixtures to improve the solubility and/or lower its volatility, i.e., reduce the evaporation rate [27].

In this study, dimethyl sulfoxide (DMSO) was selected. Despite its very high boiling point (189 °C), it is recognized as a low-toxicity solvent [11,28]. During electrospinning, DMSO allows for a much longer process (time duration), without solidification at the injector (needle) tip [29]. But the main advantage of dimethyl sulfoxide is that high fiber quality is obtained leading to a well-defined morphology [12,30]. In many cases, electrospinning with DMSO is carried out by coupling polymers or mixing solvents [13,29,31]. Indeed, the lignin of *Miscanthus x giganteus* has already been the subject of an electrospinning study in which a coupling with polyacrylonitrile was used in N, N-dimethylformamide as solvent. The purpose of using polyacrylonitrile was to achieve a carbon fiber with viable mechanical properties [31]. In the use of dimethyl sulfoxide, there are many studies employing it with various solvents in order to improve its evaporation for better removal of residual solvent and energy savings. However, the addition of another solvent may have a higher environmental impact. In general, it was necessary to use an additive in the solvent to obtain good-quality lignin fibers via electrospinning [9,11]. These additives are usually polymers to control the physico-chemical properties of the solutions, but the use of toxic solvents can limit the desired ecological aspect [17].

Electrospinning of a polymer solution is determined by three groups of parameters: solution parameters (concentration, conductivity, and surface tension), processing parameters (voltage, flow rate, needle, and collector distance), and environmental conditions (humidity and temperature) [6,17]. Variability in these parameters is at the origin of the sensitivity of the electrospinning process [7,11]. Although all the parameters are to be considered, the main parameter is the polymer concentration [11,12]. The concentration must be above a specific value to stabilize the jet (viscosity and elasticity) [12]. A high concentration also promotes an increase in the fiber diameter [3,12]. On the other hand, a low concentration promotes the formation of beads [11,12,26]. The solution concentration also has an important effect on electrospinning because it controls the interactions between the molecular chains, which determines the rheology controlling the degree of stretching and the morphology of electrospun fibers [3,32]. Another factor, the electrical conductivity, has a significant influence on the process as it directly affects the fiber structure: smooth or beaded (non-homogeneous) [33]. Increasing the electrical conductivity of the lignin solution leads to the accumulation of charges on the surface of the jet, which is beneficial during its stretching [3,34]. Finally, surface tension controls the deformation and evaporation of the solution [7,30]. The evolution of these parameters is closely linked to the nature of the solvent.

Among the processing parameters, the voltage has an important effect because a minimum voltage is required to induce the formation of charges on the surface of the droplet to overcome the surface tension and form a Taylor cone [3,29]. The applied voltage affects the morphology of the electrospun fiber because it determines the electrostatic repulsion between the charges and the interaction between the jet and the external electric field [3,29]. High applied voltage promotes jet elongation and the right orientation of lignin molecules, but exerts a minimal effect on the diameter of electrospun lignin fibers [3,35]. On the other hand, a very high voltage can lead to fiber break-up [35]. This work is a continuation of the research carried out within our laboratory on the development of bio-sourced carbon fiber from an Organosolv lignin produced using an internal process.

The general objective of this study is to produce, by electrospinning and based on an Organosolv, lignin from *Miscanthus x giganteus*, a lignin fiber suitable for high-added-value applications. Electrospinning is selected to generate nanofibers using DMSO as a sole solvent because of its low toxicity and no other polymer additives are used except organosolv lignin solution in DMSO. An experimental study is performed to determine the best set of processing parameters to achieve good (without beads) and stable (constant and small diameter) lignin fibers, by electrospinning Organosolv Miscanthus lignin solutions in DMSO.

## 2. Materials and Methods

A three-step methodological approach was carried out as presented below. In the first phase, lignin was extracted from *Miscanthus x giganteus* by the catalytic Organosolv process. The Organosolv lignin obtained was then characterized by Fourier Transform Infrared Spectroscopy (FTIR) and X-ray photoelectron spectroscopy (XPS), while its purity was evaluated by the Klason lignin content. The hydrolysate, obtained after lignin precipitation and filtration, was characterized by High-Performance Liquid Chromatography (HPLC).

The second step of this experimental work consisted of the solubilization in DMSO of the lignin obtained. Lignin was solubilized at different concentrations. The lignin solutions in dimethyl sulfoxide were characterized by the following three parameters: electrical conductivity, viscosity, and surface tension.

The third step was the electrospinning of the different solutions. We varied the main process parameters to achieve the best conditions for each solution analyzed. The electrospinning of these solutions enabled the production of lignin fibers which were analyzed to determine their quality and size (diameter) distribution.

### 2.1. Organosolv Lignin Isolation from Miscanthus x giganteus

**Figure 1:**
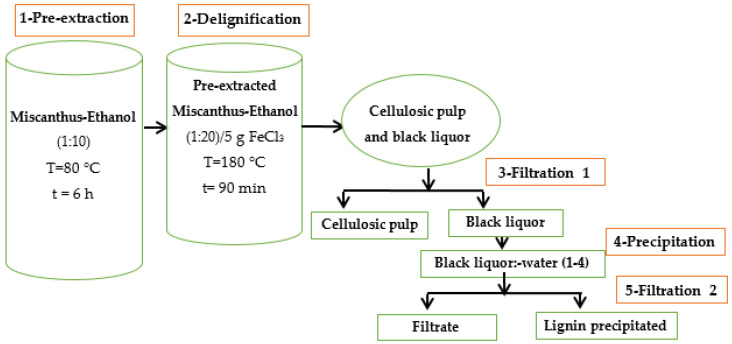
Schematic representation of the Organosolv process.

Lignin isolation was carried out on *Miscanthus x giganteus* obtained from a fall 2022 harvest in the Ontario region of Canada and provided by DMT BioProduct (Aylmer, ON, Canada), a start-up company specializing in bioproducts from *Miscanthus x giganteus*. A solvent (aqueous ethanol 50%): biomass mixture ratio of 1:20 was selected, with the addition of (FeCl_3_) as a catalyst (5% of the mass of the dry biomass). The equipment is made of two reactors: the first one is a Glace Co 4 L type for pre-extraction, while the second is a 2 L PAAR series 4842 for biomass delignification. After air drying the pre-extracted biomass for about ten days, the lignin was isolated according to the method described in Figure 1.

The isolation of this lignin was carried out in four main steps. The first is a pre-extraction intended to eliminate extractable compounds as much as possible by a water-ethanol mixture (50%). The pre-extraction carried out in the first reactor mentioned above, required 6 h at a temperature of 80 °C. The second stage consists of the delignification of the lignocellulosic material. It was carried out in the second reactor. During this stage, we proceeded with a mixture of pre-extracted *Miscanthus*-Ethanol (1:20) with a small amount of iron III chloride, representing 5% of the dry biomass mass. The whole mixture introduced into the reactor was left to cook for 90 min at 180 °C.

After delignification, we obtain a mixture of cellulose paste and black liquor. The mixture was separated by a simple filtration which is the third step in the process. During this stage, the black liquor is collected separately from the cellulosic pulp. The fourth process step was carried out by precipitating the lignin contained in the black liquor by mixing it with water in a 1:4 ratio. Finally, the fifth step, which represents a second filtration, consists of the separation of the lignin from the filtrate containing the sugars removed during the pulping process.

### 2.2. Characterization of the Miscanthus x giganteus Organosolv Lignin

Different physicochemical properties of the obtained Organosolv lignin were determined as follows.

#### 2.2.1. Lignin Purity

The lignin purity was determined by a Klason lignin analysis according to ACNOR G-8, TAPPI T 13m-54, and ASTM D-1106 [36]. All the experiments were repeated 4 times to evaluate the purity of the lignin obtained.

#### 2.2.2. HPLC Analysis of the Filtrate from Klason Lignin

HPLC analysis was performed on an Agilent HPLC 1200 series (Agilent Technologies, Germany equipped with a Rezex RHM-Monosaccharide H+ (8%) (300 mm × 7.8 mm) column. This analysis was performed on the filtrate from the Klason lignin determination in the Organosolv lignin, to reveal the sugars remaining in the Organosolv lignin. The filtrate was neutralized to pH = 7 with calcium carbonate and then filtered to 0.2 µm before analysis. The analysis was repeated three times according to the NREL standard (National laboratory of the U.S. Department of Energy) [36].

#### 2.2.3. Quantification of Nitrogenous Matter

The amount of nitrogenous matter was determined by assessing the nitrogen and therefore the protein content of the lignin obtained. This analysis was carried out using a Perkin Elmer (Wellesley, MA, USA) Precisely Serie II nitrogen analyzer 2410.

#### 2.2.4. Polymer Properties

The polymer properties of the *Miscanthus x giganteus* Organosolv lignin were studied via gel permeation chromatography (GPC). Sample preparation involved weighing 20 mg of lignin and adding 2 mL of tetrahydrofuran (THF) to solubilize it. The mixture was stirred for one hour before filtering through a 0.45 µm syringe filter and finally placed in the analyzer. The analyses were performed on an Agilent (Santa Clara, CA, USA) HPLC 1200 series equipped with a diode detector (DAD) and a 5 m Mixed-D 300 mm × 7.5 mm gel PL column. The column was operated at 50 °C with THF at a flow rate of 0.5 mL/min. The column was calibrated using polystyrene (580–28,770 Da, Agilent, Germany).

#### 2.2.5. FTIR Analysis of *Miscanthus x giganteus* Organosolv Lignin

The normalized FTIR spectrum of the isolated Organosolv lignin was obtained using a spectrometer (ATR-FT-IR/FT-NIR Spectrophotometer 400, PerkinElmer, Shanghai, China). The spectra were obtained from 64 scans and collected for wavenumbers ranging from 4000 to 650 cm^−1^ with a resolution of 4 cm^−1^.

#### 2.2.6. XPS Analysis of the *Miscanthus x giganteus* Organosolv Lignins

The chemical composition of the Organosolv lignin surface was investigated by X-ray photoelectron spectroscopy (XPS) using a PHI 5600-ci spectrometer (Physical Electronics, Eden Prairie, MN, USA). The main XPS chamber was maintained at a low pressure (less than 1 × 10^−8^ Torr). A standard aluminum X-ray source was used to record survey spectra and high-resolution (HR) spectra. Survey analyses were performed with charge neutralization, while HR analyses were carried out without charge neutralization. The detection angle was set at 45° with respect to the normal of the surface and the analyzed area was 0.05 cm^2^. The curve fitting procedure of HR spectra was performed by means of a least-square minimization procedure based on Gaussian–Lorentzian functions and a Shirley-type background. The C1s peaks were referenced at 285 eV (C-C and C-H).

### 2.3. Solubilization of Lignin in DMSO

Using an increasing concentration approach, we gradually increased the lignin concentration to reach a fiber start of approximately 55%. From this concentration, we retained for analysis the concentrations studied and presented in this work. Indeed, the solubilization of lignin was carried out for different concentrations: 55, 60, 62, and 63% by weight. These concentrations were achieved by leaving the lignin-DMSO mixture in an Isotemp 22 type bath at 90 °C for 8 h. Once the lignin was completely solubilized, the solution was left to rest for 1 day before electrospinning.

### 2.4. Analyses of the Polymer Solution

The lignin solutions’ viscosity, surface tension, and electrical conductivity were studied by the following methods:

#### 2.4.1. Viscosity

Viscosity analysis was carried out using a cone and plate (diameter = 50 mm, angle = 4°) geometry on an ARES rheometer (TA Instruments, Montreal, QC, Canada). The analyses were conducted at room temperature which is the same as for the electrospinning runs. Then, frequency sweeps were performed in the linear viscoelastic regime to determine the complex viscosity as a function of frequency for each sample.

#### 2.4.2. Surface Tension

Surface tension was determined by the pendant drop method using a goniometer FTA 200 from First Ten Angstroms (Newark, CA, USA). A total of four repetitions were performed for each sample.

#### 2.4.3. Electrical Conductivity

The electrical conductivity was determined for studied DMSO solutions, using the YSI (Yellow Springs, OH, USA) Model 35 conductance meter. A total of four measurements were carried out to get an average and standard deviation.

### 2.5. Electrospinning of Lignin Solutions

**Figure 2:**
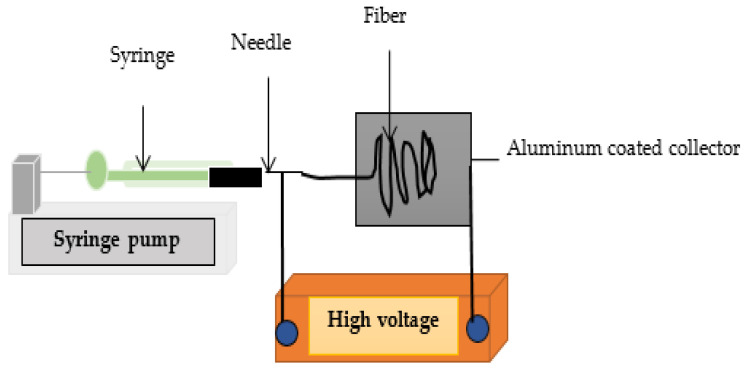
Schematic representation of the electrospinning setup.

Electrospinning was performed in the horizontal mode. The setup is composed of a Jensen Global 22 Gauge Blunt needle and a 1 mL Henke Sass Wo syringe. The syringe was mounted on a pump controlling the feed rate. A metal plate covered with non-stick aluminum foil (Saguenay, QC, Canada) served as the collector. A Plexiglas box with an opening for needle placement was connected to a Spellman CZE1000R high-voltage source (Hauppage, NY, USA), see Figure 2. A connection was made to the voltage source terminals at both the needle and collector ends to ensure the flow of electric current. The following electrospinning parameters were studied: flow rate (0.1–3 mL/h), needle-collector distance (5–30 cm), and voltage (5–30 kV). The experiments were carried out (measured, but not controlled) under ambient humidity (40%) and temperature (24 °C).

The lignin concentrations in DMSO are fundamental variables of our analysis. The selection of process parameters, such as flow rate, distance between the needle and the collector, and voltage, was carried out through an experimental approach based on the quality of the obtained fibers.

### 2.6. SEM-EDX Fiber Analysis

After electrospinning, the lignin fibers were observed using a JEOL JSM-6360LV (Tokyo, Japan) scanning electron microscope (SEM) coupled to energy-dispersive X-ray spectroscopy (EDX) to determine the quality of lignin fibers, and to check if residual sulfur was present. To determine the fiber diameter distribution, the Image J software version 1.53d was used. The images were randomly selected and a minimum of 50 fibers were used for the statistics.

## 3. Results and Discussion

### 3.1. Purity Status of Organosolv Lignin from Miscanthus x giganteus

Lignin from *Miscanthus x giganteus* from the Organosolv process catalyzed with (FeCl_3_), was obtained with a recovery rate of 52.2%, which is higher than for similar studies dealing with lignins isolated from herbaceous plants via an Organosolv process [37,38]. For example, a recovery rate of 25.1% using 50% ethanol from an Organosolv process on *Miscanthus x giganteus* was reported [37]. One explanation for this difference could be the catalyst used and its concentration. The Klason lignin content of the Organosolv lignin of *Miscanthus x giganteus* was determined to be 89.3% with about 10% impurity. Hamzah et al. obtained much less purity (51.6%) for ethanol-extracted Organosolv lignin from *Miscanthus x giganteus* (50%), which can be explained by the different processing parameters and catalyst used, as well as the origin of the plant and its harvest period [37]. The impurities were mainly proteinaceous matter related to the origin of the species and not to the quality of the process [37,38].

In our case, the impurities are about 6.65% sugars, mainly glucose (4.49%), as well as fructose (1.22%), rhamnose (0.22%), and cellobiose (0.62%). Ashes made up about 2% of these impurities. Glucose is usually very resistant to even the most severe pre-treatment conditions, which explains its higher residual content. In electrospinning, glucose is not very disturbing because it is the main element in the production of cellulose fibers [39,40].

Based on these findings, impurities from the nitrogenous material could be the limiting factor in the electrospinning of this lignin. Analysis of the nitrogenous material revealed that this lignin contains about 1% of nitrogen which corresponds to nearly 6% of protein. The lignin purity here is lower compared to other studies using the same process [7,24,37]. The difference can be explained by the type of species used. In most previous studies, the lignins were isolated from forest species. With almost 90%, the Organosolv lignin purity obtained here is nevertheless considered to be enough for electrospinning and Table 1 presents the properties of the lignin obtained.

### 3.2. Lignin Polymer Properties

The organosolv lignin isolated from Miscanthus by the catalytic procedure described previously was determined to have average molecular weight by mass and the number of 1040 and 437 g/mol, respectively, leading to a polydispersity index (PI) of 2.38. This lignin has a low molecular weight compared to several others, resulting from various processes [7,24]. The impurities, in terms of nitrogen and sugars, have a very small effect on the molecular weight of this lignin, compared to Kraft and sulfite lignins [20]. But the low molecular weight of lignins is a disadvantage for electrospinning as this parameter will affect the viscosity of the solution [24,41].

With a polydispersity index somewhat above 2, the molecules of this lignin do not seem to be much dispersed. The low PI is the origin of a spherical crowning of the molecules, which can limit its reactivity [17]. A high molecular weight and a low polydispersity index are required for the electrospinning of lignin. Beyond purity, the main limitation of the application of Organosolv lignins is their molecular weight. Miscanthus organosolv li gnin obtained in this work is no exception, it is consistent with the literature on the predicted molecular weights of organosolv lignins. Miscanthus lignin has a lower molecular weight than those obtained from softwood and hardwood species applying the same process [7,24]. The latter concluded on the favorable character for electrospinning of the organosolv lignin resulting from this process. The low molecular weights of the Miscanthus organosolv lignin determined in this research required therefore quite a high concentration of the DMSO solutions for electrospinning.

### 3.3. FTIR Analysis of Organosolv Lignin from Miscanthus x giganteus

The FTIR analysis of the Organosolv lignin of *Miscanthus x giganteus* allowed us to characterize the molecules in terms of their functional groups. Using the spectra, the characteristic peaks reveal the different distinctive elements of this lignin. A peak at 3350 cm^−1^ reveals the presence of -OH functionalities. These hydroxyl groups come from both alcohol and phenolic hydroxyl groups [42,43]. They can also be related to the residual sugars (Table 1).

Around 2900 cm^−1^, a peak relative to stretching in C-H bonds in aromatic methoxyl, methylene, and methyl groups of the side chains is observed [38]. The peak between 1700 and 1800 cm^−1^ indicates the presence of the C=O group. According to Kaur et al., this peak can be attributed to the esterification of the phenol and alcohol of the propane chain (C_α_ and C_γ_) which occurs during the pulping process [42,43]. At 1604 cm^−1^, a peak confirms the presence of N-H protein impurity, while a peak around 1515 cm^−1^ represents a guaiacyl-type aromatic ring [42,43].

Between 1452 and 1406 cm^−1^, a peak corresponding to -C-C- bonds is associated with the link between the aromatic rings. This peak can be related to the polymerization of aromatic rings during processing or the native structures of lignin [42,44]. The peaks at 1113 and 1028 cm^−1^ indicate the presence of the guaiacyl and syringyl units of lignin, respectively. The peak at 830 cm^−1^ represents C-H bending in syringyl units [42,43]. The results obtained are consistent with the literature, especially the study of Bergs et al. on the analysis of the structure of lignins from *Miscanthus x giganteus* [39].

The FT IR analysis spectrum of the organosolv lignin from *Miscanthus x giganteus* demonstrated the absence of non-biological molecular compounds. Contrary to the same analysis performed on lignin from *Miscanthus* obtained through a non-organosolv process, the latter shows the presence of metal-sulfur-type interactions at the surface of carbon around 607 cm^−1^ [30].

### 3.4. XPS Analysis of Organosolv Lignins

**Figure 3:**
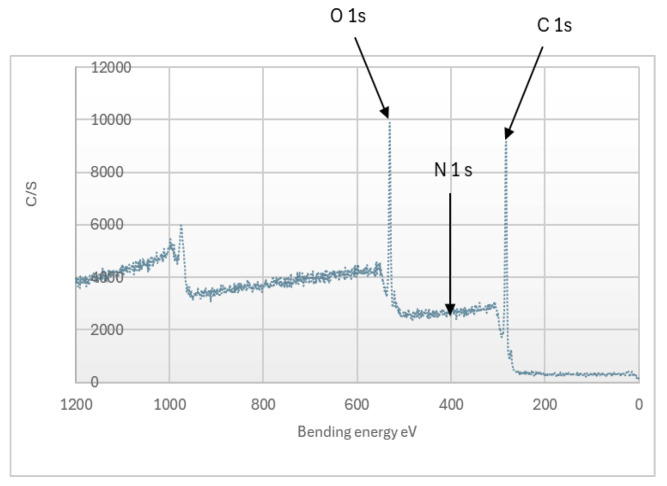
XPS spectrum Organosolv lignin from *Miscanthus x giganteus*.

Figure 3 shows that the lignin obtained is composed of: 74.2% carbon, 24.8% oxygen, and 1% nitrogen. The nitrogen content is consistent with the results from the tests of lignin purity (Table 1). According to Stark et al. [45], the elements of interest are carbon (C1s), oxygen (O1s), nitrogen (N1s), sodium (Na1s), and sulfur (S1s and S2p) [45]. The XPS analysis reveals that the lignin is free of sulfur and sodium, while only a small trace of nitrogen is observed apart from carbon and oxygen.

**Figure 4:**
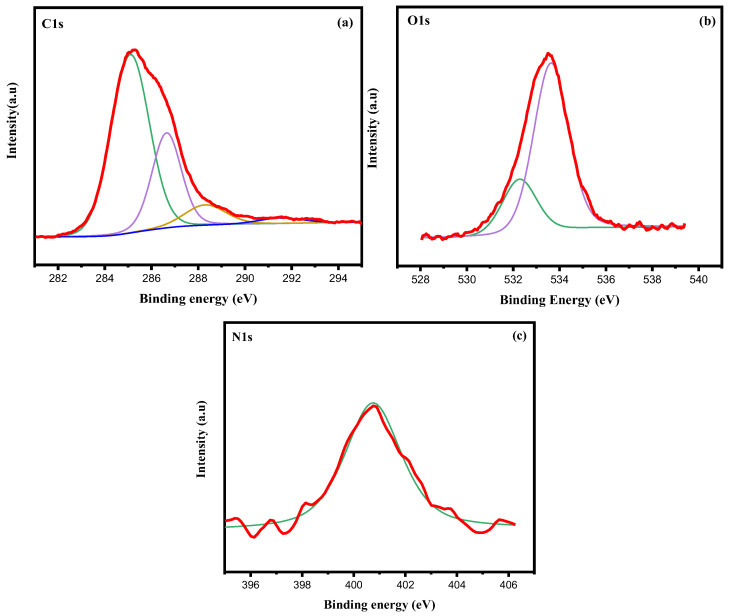
XPS spectrum (deconvolution) of (**a**) C1s, (**b**) O1s, (**c**) N1s.

The bond energies between different atoms allow us to determine the type of functional groups. The C1s peaks are composed of four peaks 284.95, 286.49, 288.75, and 291.60 eV, corresponding to carbon atoms including different bonds as C-C/C-H, C-O, C=O, and N-C, respectively (Figure 4) [29,41]. O1s is composed of two peaks at 532.20 and 533.50 eV corresponding to functional groups O-H and O-C [29]. At the nitrogen level, a bond energy of 400 eV indicates the presence of C-N- groups [41].

The XPS analysis also confirms a high amount of C-O- bonds compared to C-C bonds (Figure 4a–c). This indicates that the lignin obtained by the Organosolv process catalyzed with FeCl_3_ is not denatured, keeping a high amount of ether (C-O-C) bonds, very important in native lignins.

**Figure 5:**
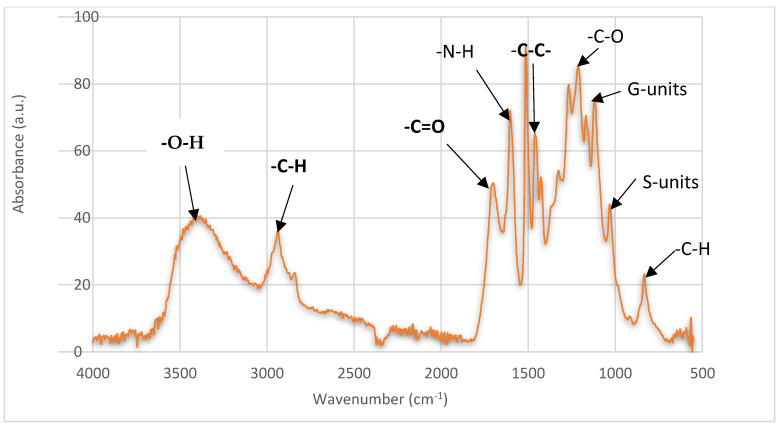
FTIR spectrum of the Organosolv lignin from *Miscanthus x giganteus*.

The functional groups observed are in agreement with the FTIR spectrum (Figure 5).

### 3.5. Properties of Lignin Solutions

By dissolving the lignins in DMSO without additives, homogeneous solutions were prepared at different concentrations (55, 60, 62 and 63%). This range of concentration confirmed the good solubility of Organosolv lignin in dimethyl sulfoxide. The solutions were analyzed for viscosity, electrical conductivity, and surface tension, the main parameters involved in the electrospinning performance. The dissolution of the obtained lignin was favored in DMSO due to its high purity and composition, which was essentially made up of biological molecular compounds. Indeed, polymer solutions can be diluted, semi-diluted, or concentrated [46]. The obtained solutions presented the following situations: diluted and concentrated. For concentrations of 55, 60, and 62%, the solutions appeared diluted, while the solution at 63% seemed genuinely concentrated. These two states show differences in the properties of solutions.

#### 3.5.1. Viscosity

**Figure 6:**
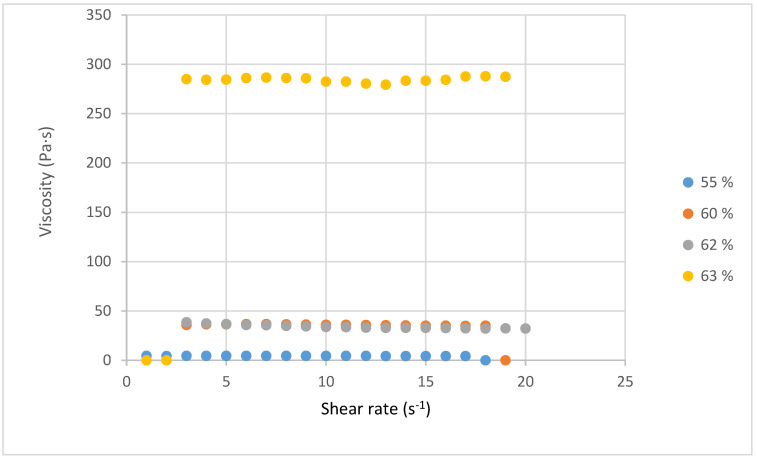
Viscosity of the Organosolv lignin in dimethyl sulfoxide at different concentrations.

Figure 6 presents the viscosity curves for four lignin solutions. In all cases, the values are constant indicating Newtonian behavior.

Starting from a viscosity of 2.0 Pa·s at 0% lignin, we notice an increase in viscosity depending on the amount of lignin added [28]. For the 55% solution, a viscosity of 4.3 Pa·s was determined, while the viscosity increased to 35.15 Pa·s at 60%. This represents almost a 10-fold increase in viscosity for only a 5% increase in concentration. Although a limited viscosity variation is observed between 60 and 62, a major increase (up to 284 Pa·s) is observed when the concentration reaches 63%. Huang et al. justified this significant viscosity increase by increasing molecular entanglement with concentration. They also stated that a high amount of lignin led to a higher extent of the condensation reaction inside the lignin, affecting the molecular weight and rheological properties [26]. Akbari et al. [47] reported that the entanglement of molecular chains with the increase in concentration would be the cause of the increase in viscosity. They found a 10% increase in viscosity for just a 1% increase in concentration [47]. In our study, we observed three stages of viscosity evolution; these stages present a different variation in viscosity. An increase between 55 and 60%, and between 60 and 62%, we have a quasi-constancy of viscosity.

At the concentration of 63%, a very significant increase in viscosity is recorded. The interactions between the Organosolv lignin and dimethyl sulfoxide may be the cause of this phenomenon. Indeed, the 63% solution is very concentrated; this level of concentration can lead to a more significant entanglement of molecular chains, which could cause this variation beyond all expectations. Therefore, a question remains regarding the third stage of viscosity evolution for the concentration of 63%, whether the interactions between molecular chains explain the evolution of viscosity, while this increase remains to be further studied. At this level of solution concentration, a change in concentration of 1% can allow a transition from one situation to another. Albari et al. have shown in their electrospinning studies, that an increase of 1% in concentration allowed for a transition from an electrospinnable solution to a non-electrospinnable one [47].

In electrospinning, the viscosity is highly important because a low viscosity leads to bead formation, while a high viscosity delays the formation of Taylor cones and decreases the deformability of the jets, as well as the possibility of gel formation preventing the formation of nanofibers [29,48]. In view of these results, the ideal viscosity for the solubilization of *Miscanthus organosolv* lignin in DMSO is around 62% generating a viscosity of 35 Pa·s.

#### 3.5.2. Electrical Conductivity

Measuring the electrical conductivity of the solutions allowed us to determine the importance of lignin concentration for this property. Table 2 shows the evolution of electrical conductivity as a function of the concentration of lignin solution in DMSO. It shows that at 0%, the conductivity is 3 µS/cm, while at 55%, we obtain an electrical conductivity of 0.95 µS/cm, and at 60%, it is 0.26 µS/cm. It then decreases slightly to 0.16 and 0.11 µS/cm at 62% and 63%, respectively. The ideal solution is around 0.16 µS/cm and corresponds to a concentration of around 62%. This electrical conductivity, although not very high, allows the formation of good fibers with uniform diameters as described later. High conductivity helps to limit the concentration of the polymer as mentioned in the literature [46]. However, in the case of our study, the solution with the highest electrical conductivity was the one that presented the occurrence of most pearls, despite a lower concentration.

The low conductivity of the solutions is due to the high lignin concentration in the solution. A high concentration is necessary due to the low molecular weight of this lignin. Studies using biopolymers usually determine very low conductivity. This is why additives are used for electrospinning [7,49]. Electrical conductivity is affected by the type of polymer and solvent, the concentration of the polymer, and the temperature [29]. The solution studied showed a decrease in electrical conductivity with an increase in lignin concentration. We can observe in this study that electrical conductivity is dependent on lignin concentration. According to Denhad et al. [29] when a polymer has ionic functionalities, the electrical conductivity of the solution depends on the concentration of the polymer. The variation in the electrical conductivity of the obtained solution is justified [29]. The impact of the solvent on the electrical conductivity of the solution seems more important than that of the polymer. With a conductivity of pure DMSO measured as 3 µS/cm, it decreases significantly as the concentration of lignin increases [28]. Despite the very low solution conductivity of lignin solutions in DMSO, it was possible to electrospin these solutions as described. DMSO is a polar solvent, but aprotic, which means not available for hydrogen bonding. Its low conductivity is a challenge in electrospinning experiments, especially when using a polymer of low polarity such as lignin. Therefore, the final result achieved in this study is quite important as good-quality lignin fibers were obtained with little remaining solvent. The molecular interactions between lignin polymer and DMSO remain to be further studied.

#### 3.5.3. Surface Tension

Table 2 shows that the surface tension of the lignin solutions decreases with increasing lignin content. With a surface tension of 43.53 mN/m, DMSO is a relatively low volatility solvent and the low surface tension obtained does not promote fast evaporation [28]. This can lead to the presence of residual solvent in the fibers [13,33]. In this study, for a spinning time of 1 h, a direct relation was observed between the surface tension of the solution and the residual solvent in the fibers. This study shows that the applied voltage predominantly outweighs the surface tension of the solution. Indeed, for the successful electrospinning of a solution, it is necessary that the forces opposing the initiation of the jet, mainly the surface tension, be dominated by the applied voltage, which leads to the formation of the Taylor cone [7,29].

The increase in concentration showed a decrease in the surface tension of the solution. The evolution of surface tension as a function of increasing concentration has helped to mitigate the formation of beads in our study. This decrease in surface tension not only allows for the formation of a stable jet, but also eliminates the beads observed at the 55% solution, which corresponds to the highest value of surface tension. The surface tension around the 62% concentration solution seems to be ideal for the electrospinning of this lignin. According to Xie et al. [50], the formation of beads in electrospun fibers can be caused by the surface tension of the charged jet which would transform the jet into droplets [50]. This phenomenon was observed for the lignin solutions at concentrations of 55 and 60% concentration, but not in those of 62 and 63%.

### 3.6. Properties of the Electrospun Lignin Fibers

The electrospinning of the Organosolv lignin from *Miscanthus x giganteus* was carried out with DMSO as the solvent to study the effect of different processing parameters: the needle-collector, applied voltage, and flow rate, while the humidity and temperature (environmental conditions) were kept constant at 40% and 24 °C. Through the electrospinning tests carried out and using EDX-SEM analyses, it was possible to observe both the evolution of the electrospun fiber and the effect of the solvent.

#### 3.6.1. Effect of Lignin Concentration on Fiber Diameter

**Figure 7:**
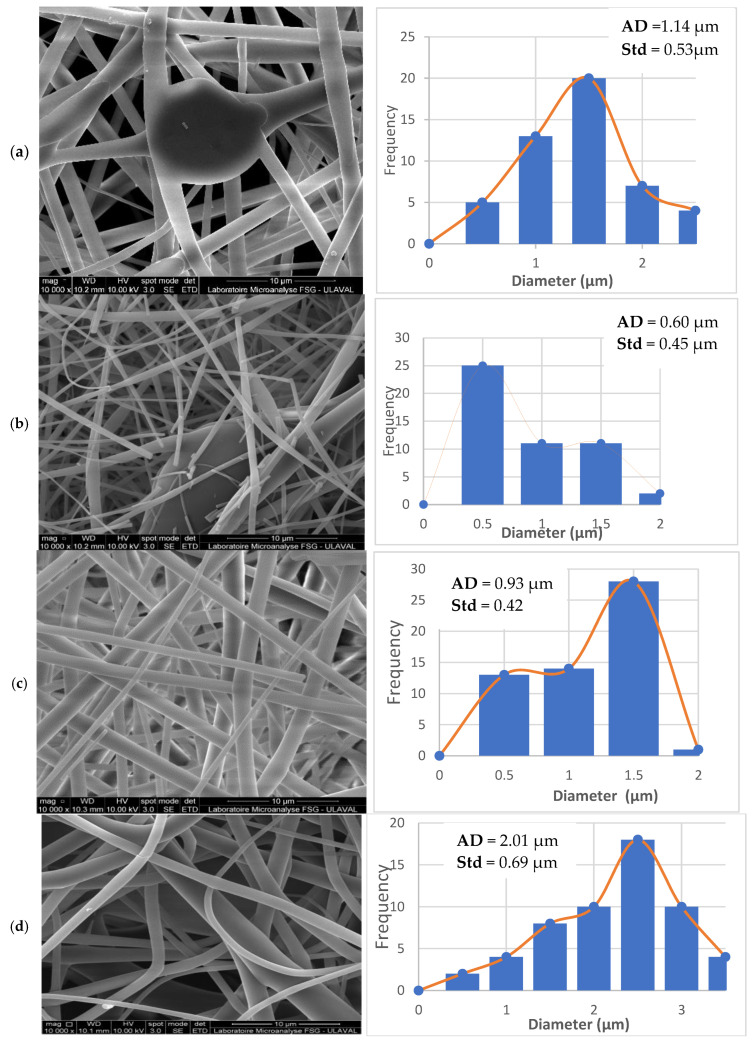
SEM image and diameter distribution of lignin fibers from solutions (**a**) 55%, (**b**) 60%, (**c**) 62% and (**d**) 63%.

The solution concentration is an important parameter in electrospinning as it changes the type of fibers formed. In preliminary trials, concentrations above 63% were too viscous to run (high pressure for the injection pump), while concentrations below 55% did not produce a stable process with good fibers. Here, the fiber diameter is highly affected by the latter as higher lignin concentration produces higher fiber diameter [4,10,11]. It can be seen that at 55% (Figure 7a), good fiber formation starts with almost non-uniform dimensions and small diameters varying between 0.26 and 2.5 µm. Starting at 60% (Figure 7b), the results show that the fiber diameter increases with decreasing bead formation. The resulting fibers were determined to have diameters ranging from 0.13 to 2 µm. At 60%, these fibers are still slightly non-uniform and mainly have a diameter below 0.5 μm. Although very small, the presence of residual solvent requires a higher lignin concentration.

At 62% (Figure 7c), the fiber diameter varies between 0.44 and 2.5 µm. This solution yielded fibers dominated by a diameter between 1 and 1.5 μm. But at 63% (Figure 7d), larger diameters are produced up to 3.2 μm. The distribution of fiber diameters confirms that it is a function of concentration. This trend is also confirmed in the literature [11,42].

According to the measurements of the average diameters of the obtained fibers, determined by a statistical approach based on image analysis, a different evolution regarding the category of fibers can be observed (Table 3). The fibers’ diameter exhibited a lack of uniformity based on the categories, which was mainly due to the presence of beads on some fibers. In fact, the presence of pearls in the fibers generally comes from low polymer concentration [11]. Our study showed that for the lowest concentration, namely 55%, we have more fibers with beads for a lower viscosity, which is translated into a lower entanglement of the molecular chains Indeed, the concentration of the solution profoundly affects the morphology of the fibers, thus, less entanglement of the molecular chains and therefore the formation of beads occurs in fibers at low lignin concentrations [42]. According to Gupta et al., the properties of the solution, as well as the nature of the solvent or the polymer-solvent interactions, are the factors that affect most significantly the diameter of lignin or carbon fibers [25]. This assertion by Gupta et al. shows that the quality of the obtained fibers (morphology, diameter, bead, and others) mainly depends on the properties of the solution, even though process parameters such as flow rate, needle-collector distance, and voltage are also determined. These last parameters adjust an electrospinnable polymer-solvent mixture.

In view of the purity quality of the studied organosolv lignin, which is shown to be conducive to electrospinning, we still need to better understand the impact of the solvent used [7,23,24]. Here, a relationship between the presence of beads and the residual sulfur from the solvent is observed. It is also reported that the number of beads decreases with increasing polymer concentration. But there is a limit for which the viscosity becomes too high for stable processing [39]. Overall, optimal results at different lignin concentrations were obtained from: a distance of 20 cm, a flow rate of 0.3 mL/h, and a voltage of 25 kV. These conditions resulted from an optimal concentration of 62%.

#### 3.6.2. Effect of DMSO on the Sulfur Content of Lignin Fibers

The electrospun fibers were produced using DMSO as a solvent. The effect of the solvent used in the electrospun lignin fibers was investigated by SEM-EDX fiber analysis. It was necessary to determine the presence of residual sulfur in the lignin fibers obtained and the results are presented in Table 4. It was found that the fibers from the 55% lignin solution contained 4.40% sulfur and that this value decreased with increasing lignin concentration. This trend can be explained by the fact that increasing the lignin content decreases the DSMO concentration. This implies that a decrease in solvent content leads to less residual sulfur in the fibers.

Another explanation could be the time required for the solvent to evaporate. In fact, DMSO has a fairly high evaporation temperature, limiting its complete removal during processing. Increasing the lignin concentration generated solutions of higher viscosity, which during the electrospinning process requires a longer time for the Taylor cone formation and solution injection, which could explain the lower sulfur content with higher concentration solutions. According to Koshe et al. [12] the use of DMSO has a great impact on fiber quality as it can prevent the formation of ribbon-like fibers. The solvent is a very important parameter in electrospinning, as it can be decisive for the quality of the fibers obtained. In addition to preventing the formation of ribbon-shaped fibers, DMSO can promote the development of smooth fibers [12]. In terms of the quality of the fibers obtained, we noticed the formation of smooth fibers at the solution level of 62%, (Figure 7c). Beyond the appearance of beads, we do not have fibers in the shape of ribbons. The only disadvantage of using DMSO remains the appearance of residual sulfur in the obtained fibers. Khan et al. noted the presence of DMSO in the lignin fibers after electrospinning and highlighted the possibility of drying them through carbonization in order to eliminate the residual solvent [12]. Lignin is naturally free from sulfur; the latter can only come from the solvent. It is interesting that the increase in lignin concentration in DMSO solution is accompanied by a decrease in sulfur content in electrospun fibers, indicating an entanglement of lignin polymer chains, thus prolonging the electrospinning process and consequently improving solvent evaporation, which resulted in less residual sulfur in the lignin fibers (Table 4) [48].

### 3.7. FT IR Analysis of Lignin Fibers

**Figure 8:**
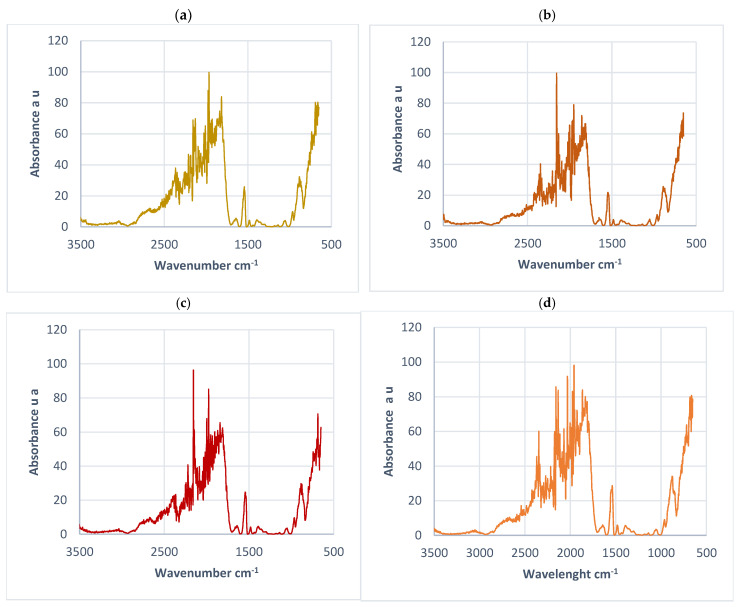
FTIR spectra of electrospun lignin fibers (**a**) 55%, (**b**) 60%, (**c**) 62%, (**d**) 63%.

The comparative analysis of electrospun lignin fibers performed by FT-IR shows differences between the initial spectrum of lignin and that of Miscanthus lignin fibers. The FTIR spectra of lignin fibers (Figure 8) show the presence of peaks at common wavenumbers around 1538, 879, and at 650 cm^−1^ for the four lignin concentrations. It is noteworthy that the peak at 650 cm^−1^ appears only in FTIR spectra of lignin fibers and not in the spectrum of original Miscanthus Organosolv lignin (Figure 3). According to the literature, a peak around 650 cm^−1^ is attributed to the presence of metal-sulfur interactions on the surface of carbon fiber derived from electrospinning of Miscanthus lignin [31]. Thus, the FTIR analysis confirms the presence of sulfur in lignin fibers, in harmony with what was found by the SEM-EDX analysis performed on Miscanthus lignin fibers obtained by electrospinning. It is interesting to note that the peak at 650 cm^−1^ (attributed to residual sulfur) seems to be present only in the FTIR spectra of electrospun lignin fiber and in origin organosolv lignin and it is more significant for the lignin fiber obtained by electrospinning from the least concentrated lignin solution in DMSO (55%), thus confirming the same trend as obtained by SEM-EDX analyses (Table 4). This sulfur detected in lignin fibers, according to the results of the FT IR and XPS analyses, can thus be related to the solvent DMSO residue.

## 4. Conclusions

In this work, high purity (90%) lignin was obtained from *Miscanthus x giganteus*, a herbaceous plant, by a catalytic Organosolv process, which was essential for its electrospinning. The resultant organosolv lignin was determined to have a carbon content of 72.2% with 24.8% oxygen and a low nitrogen content (1%).

The isolated lignin, although of high purity, was of low molecular weight. It showed a very good affinity with DMSO, which made it possible to obtain very concentrated solutions ranging from 55 to 63%. The rheological parameters of the solutions studied were found to be highly variable in terms of electrical conductivity (0.95–0.11 µS/cm), surface tension (27.2–20.8 mN/m), and viscosity (4.3–284 Pa·s). Depending on the processing conditions, it was possible to produce lignin fibers by electrospinning at all studied concentrations of the lignin solutions, with limitations in terms of fiber diameter (0.32–3.22 µm) and residual sulfur content (4.40–1.87%). An improvement in conductivity would be desirable, but in an effort to preserve a biobased character, it is difficult to find a green additive to improve this value.

In view of the evolution of the diameter of the lignin fibers and the effect of DMSO on the electrospun fibers, it can be concluded that electrospinning of the Organosolv lignin obtained from *Miscanthus x giganteus* was feasible, even without any polymer additives or combination with other solvents. The molecular interactions between lignin and DMSO remain to be further clarified.

## Figures and Tables

**Table 1:** Properties of the Organosolv lignin extracted from *Miscanthus x giganteus*.

Lignin Purity (%)	Impurities	Amount (%)
89.3 ± 1.2	Glucose	4.49 ± 0.20
	Fructose	1.22 ± 0.06
	Rhamnose	0.22 ± 0.01
	Cellobiose	0.62 ± 0.01
	Ashes	2.04 ± 0.02
	Nitrogen	0.91 ± 0.01
	Proteins	5.67 ± 0.20

**Table 2:** Properties of the lignin solutions in DMSO.

Lignin Concentration (%)	0	55	60	62	63
Viscosity (Pa·s)	2.0	4.3	35.2	32.2	284.3
Electrical conductivity (µS/cm)	3.0	0.95	0.26	0.16	0.11
Surface tension (mN/m)	43.53	27.2	24.1	22.5	20.8

**Table 3:** Average diameter of lignin fibers spun from different solution concentrations.

Solution of lignin fiber (%)	55	60	62	63
Average diameter (µm)	1.14 ± 0.53	0.6 ± 0.45	0.93 ± 0.42	2.01 ± 0.69

**Table 4:** Sulfur content of the lignin fibers electrospun from DMSO solutions.

Lignin concentration in DMSO (%)	55	60	62	63
Sulfur concentration in the fibers (%)	4.40	3.20	3.14	1.87

## Data Availability

Data supporting the reported results can be found in the databases of our laboratory equipment at the Renewable Materials Research Center. One can also find them in the databases of the authors of this study.
